# Crystalline structures of polymeric hydrocarbon with 3,4-fold helical chains

**DOI:** 10.1038/srep07723

**Published:** 2015-01-12

**Authors:** Chao-Sheng Lian, Han-Dong Li, Jian-Tao Wang

**Affiliations:** 1 Beijing National Laboratory for Condensed Matter Physics, Institute of Physics, Chinese Academy of Sciences, Beijing 100190, China; 2 State Key Laboratory of Environmental Criteria and Risk Assessment, Chinese Research Academy of Environmental Sciences, Beijing 100012, China

## Abstract

Molecular hydrocarbons are well-known to polymerize under pressure to form covalently bonded frameworks. Here we predict by *ab initio* calculations two distinct three-dimensional hydrocarbon crystalline structures composed of 3-fold and 4-fold helical CH chains in rhombohedral (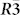) and tetragonal (*I*4_1_/*a*) symmetry, respectively. Both structures with 1:1 stoichiometry are found to be energetically more favorable than solid acetylene and cubane, and even more stable than benzene II solid at high pressure. The calculations on vibrational, electronic, and optical properties reveal that the new chiral hydrocarbons are dynamically stable with large bulk moduli around 200 GPa, and exhibit a transparent insulating behavior with indirect band gaps of 5.9 ~ 6.7 eV and anisotropic adsorption spectra. Such forms of hydrocarbon, once synthesized, would have wide applications in mechanical, optoelectronic, and biological materials.

The exploration of new technological materials is an enduring topic in high-pressure physics and chemistry^1^, and simple molecular solids are rising as potential precursors due to their high compressibility^2^. It has been shown that many unsaturated hydrocarbons form molecular crystals that polymerize under pressure into extended covalently bonded structures^3^,^4^,^5^,^6^,^7^,^8^,^9^,^10^, which can generally be recovered to ambient conditions unlike the cases in N_2_, CO_2_, and N_2_O where polymeric phases decompose at ambient pressure^11^,^12^,^13^. Ethylene^3^,^4^ and acetylene^5^,^6^ transform to linear polymers at 1–6 GPa through the opening of double and triple bonds. With the aromatic character lost, benzene polymerizes around 23 GPa in the amorphization form at room temperature^7^,^8^,^9^. A friable and colorless compound has been obtained by heating benzene above 680 K between 7 and 12 GPa^10^, while its structure is unknown so far. It is confirmed in high-pressure studies of both acetylene^6^ and benzene^9^ that laser irradiation will promote the generation of saturated network polymers with a composition close to (CH)*_n_*.

Nowadays, there is an increasing theoretical attention paid to the nonmolecular hydrocarbon crystalline phases. In addition to the well-known one-dimensional (1D) polyethylene and polyacetylene, many isomeric 2D covalently bonded hydrocarbons called graphane were suggested in the last decade^14^,^15^,^16^. Wen *et al.*^17^ found from *ab initio* calculations that benzene phases under pressure transform to ordered CH structures consisting of 1D or 2D arrays of C_6_H_6_ rings bridged by *sp*^3^ C-C bonds. This has greatly stimulated the exploration of other possible structures of polymeric hydrocarbon, which could be synthesized by applying high pressure or temperature. Using evolutionary algorithm structure searches, several 3D graphane CH structures with different graphane stackings had been reported^18^. In a recent study^19^, we predicted a diamond-like 3D cubic hydrocarbon crystal, *K*_4_-CH, through hydrogenating the hypothetical *K*_4_-carbon^20^. Subsequently, a saturated hexagonal phase of Hex-CH was also proposed from the compressed benzene^21^. It has been established that for the CH system with 1:1 stoichiometry, the fully saturated polymeric CH phases are thermodynamically more stable than any phase retaining discrete molecules^17^,^18^.

In this paper, we report by *ab initio* calculations two novel 3D hydrocarbon framework structures that comprise 3-fold and 4-fold helical CH chains in rhombohedral (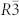) and tetragonal (*I*4_1_/*a*) symmetry, respectively, which are derived from the fully hydrogenation of our recently found all-*sp*^2^ bonded chiral carbene^22^. The structure stability, electronic and optical properties are examined up to 50 GPa. Both chiral crystalline phases are confirmed to be energetically more favorable than solid acetylene and cubane, and significantly stabilized with increasing pressure relative to the benzene II solid. The calculated vibrational and mechanical properties reveal their dynamical stability and large bulk modulus of ~200 GPa. Electronic and optical calculations show the wide-gap insulating behavior typical of saturated hydrocarbon and the unique anisotropic adsorption spectra. Our predictions can be expected to greatly stimulate future experiments to synthesize these new phases.

## Results

**Figure 1:**
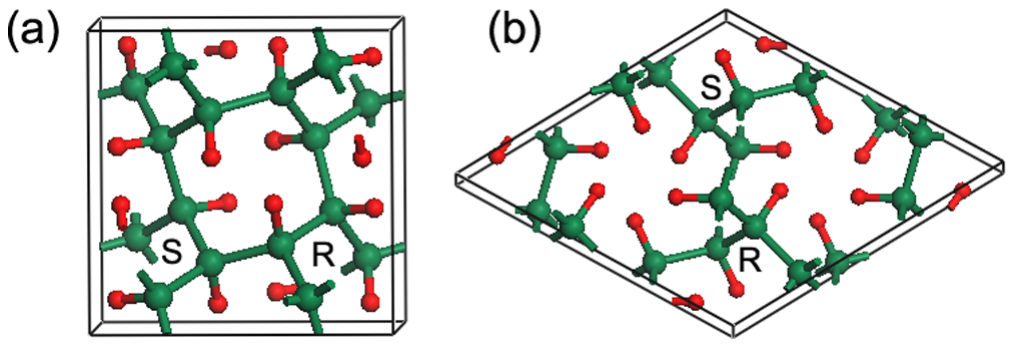
Schematic representation of the chiral crystalline structures of *T*_8_-CH (a) and *R*_6_-CH (b). Carbon atoms are shown as large olive spheres, and hydrogen atoms are shown as small red spheres. R and S refer to right- and left-handed helical CH chains, respectively.

Our newly identified chiral crystalline forms of hydrocarbon are depicted in Fig. 1. The 4-fold chiral structure [Fig. 1(a)] is body-centered tetragonal in *I*4_1_/*a* symmetry, and the optimized lattice parameters are *a* = 6.106 Å and *c* = 4.146 Å, with C and H atoms occupying 16f (0.2189, 0.1188, 0.8213) and 16f (0.0770, 0.1018, 0.6617) Wyckoff positions, respectively. This form has eight CH units in the primitive cell and we refer to it as the *T*_8_-CH. The 3-fold chiral structure in 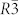 symmetry, hereafter named as *R*_6_-CH, has a rhombohedral lattice and six CH units per primitive cell. In hexagonal representation [Fig. 1(b)], its equilibrium lattice parameters are estimated to be *a* = 7.392 Å and *c* = 3.671 Å with 18f (0.4202, 0.0351, 0.0448) C and 18f (0.1816, 0.5314, 0.4693) H positions. As shown in Fig. 1, for both *T*_8_-CH and *R*_6_-CH structure, the helical CH chains are formed along the *c* axis, and each chain is connected to neighboring chains of opposite chirality (left-handed indicated by S and right-handed R) by C-C covalent bonds. The intrachain and interchain C-C bond lengths are respectively 1.559 and 1.564 Å in *T*_8_-CH, 1.561 and 1.543 Å in *R*_6_-CH, which are all close to 1.530 Å in diamond^19^. Hence, the two chiral hydrocarbons here can be considered as diamond-like CH phases similar to the previously proposed *K*_4_-CH^19^ and Hex-CH^21^; they all adopt a fully 3D framework with saturated nature of *sp*^3^ carbon.

**Figure 2:**
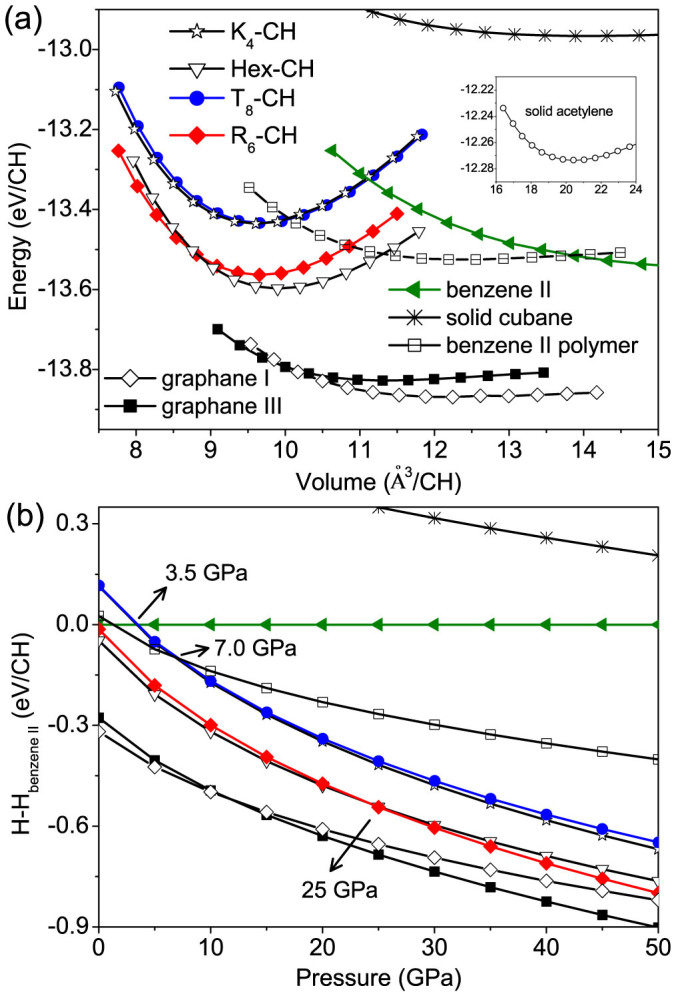
(a) Energy versus volume curves for *K*_4_-CH, Hex-CH, *T*_8_-CH, *R*_6_-CH, molecular phases of hydrocarbon such as benzene II and solid cubane, benzene II polymer, and graphane I and III. The inset shows the data for solid acetylene. (b) Enthalpy difference with respect to benzene II for the various CH phases as a function of pressure.

Figure 2(a) shows the calculated total energy versus volume curves of various hydrocarbon phases with CH stoichiometry of 1:1. We can see that the *T*_8_-CH structure is as stable as *K*_4_-CH, with energy about 0.47 and 1.16 eV/CH lower than solid cubane^23^,^24^ and acetylene^25^, respectively. Compared with *T*_8_-CH, the *R*_6_-CH structure has lower energy (close to Hex-CH), being even more stable than the benzene II^26^ crystal and its hypothetical layered polymer reported by Wen *et al.*^17^ It is noticeable that the equilibrium volumes of *K*_4_-CH, Hex-CH, *T*_8_-CH, and *R*_6_-CH are in the range 9.6–9.9 Å^3^/CH, much smaller than 14.1–20.5 Å^3^/CH of benzene II and solid cubane and acetylene (see Table I). These results suggest great potential for synthesizing the low-energy CH structures through compression of metastable molecular phases of hydrocarbon. Furthermore, we find that among all CH systems considered here the most stable phase is the layered graphane I^18^ crystal in an AA stacking (0.04 eV/CH lower in energy than graphane III). The less favorable energetic state of diamond-like CH phases compared to layered graphane is likely attributed to the stronger steric interactions of hydrogens^18^, as evidenced by the nearest H-H distances of 1.56 Å for *T*_8_-CH and 1.79 Å for *R*_6_-CH being shorter than 2.51 Å for graphane I.

To understand the pressure effect, the enthalpy difference of each phase to that of benzene II is presented in Fig. 2(b) in a wide pressure range 0–50 GPa. It is found consistent with previous calculations^18^ that instead of graphane I, graphane III becomes the most stable phase under pressures above 12 GPa. For diamond-like CH phases, an increasing stabilization with pressure can be seen relative to benzene II and the related polymer. Above 3.5 and 7.0 GPa, the *T*_8_-CH and *K*_4_-CH phases (enthalpically almost degenerate in 0–10 GPa) become more stable than benzene II and benzene II polymer, respectively. Moreover, the hexagonal phases of *R*_6_-CH and Hex-CH are more favorable than *T*_8_-CH or *K*_4_-CH in enthalpy by about 0.15 eV/CH in the whole pressure range. Meanwhile, *R*_6_-CH is found to be preferable to Hex-CH above 25 GPa, and to compete with graphane I above 50 GPa. In view of the above enthalpy results, the experimental syntheses of diamond-like CH phases are thermodynamically possible.

We have fitted the energy versus volume data to the Murnaghan equation of state^27^ to obtain the bulk moduli (*B*_0_) of different hydrocarbons, as listed in Table I. The predicted *B*_0_ of diamond-like CH phases are significantly higher than those of molecular hydrocarbons, with *T*_8_-CH and *R*_6_-CH having the values of 201.7 and 185.2 GPa, respectively. Note that for benzene II we calculate the bulk modulus to be *B*_0_ = 9.8 GPa, which is close to the reported experimental value of ~5.5 GPa^28^.

**Figure 3:**
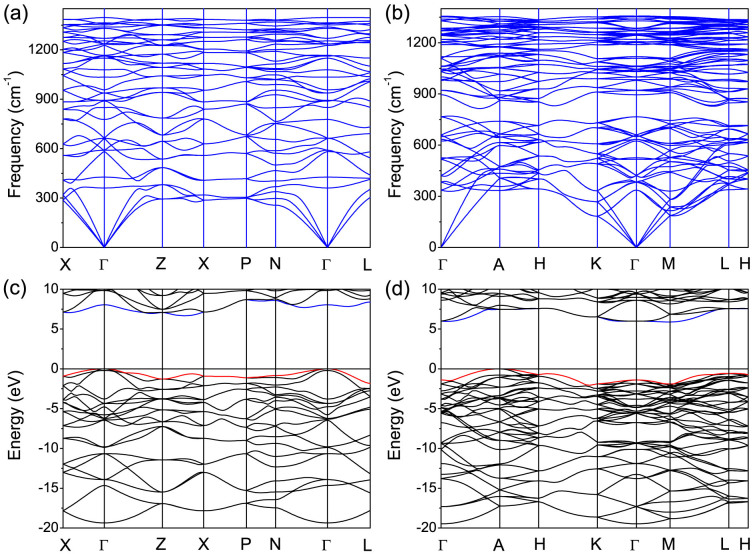
Calculated phonon and electronic band structures at 0 GPa. (a) and (b) phonon band structures for *T*_8_-CH and *R*_6_-CH. The high frequency C-H stretching modes are given in text. (c) and (d) electronic band structures for *T*_8_-CH and *R*_6_-CH.

Phonon calculations give a criterion for the structure stability of a crystal. Therefore, we calculated phonon dispersion curves for the *T*_8_-CH and *R*_6_-CH phases at 0 GPa, as presented in Fig. 3(a) and 3(b). The absence of imaginary frequency modes indicates that these two chiral structures are dynamically stable. High frequency C-H stretching phonon modes emerge around 3047 and 2992 cm^−1^ for *T*_8_-CH and *R*_6_-CH, respectively, which can be compared with the observed broad infrared peaks at 2950 and 2920 cm^−1^ (assigned to the C-H stretching modes involving *sp*^3^ carbon atoms) for amorphous samples recovered from compressed acetylene^6^ and benzene^8^,^9^. In addition, we also checked the phonon dispersion for both chiral phases under pressure confirming their dynamical stability up to at least 50 GPa.

**Figure 4:**
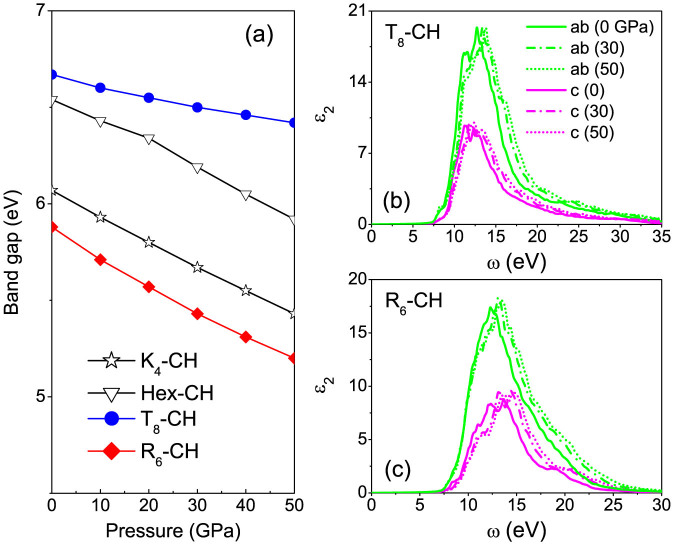
(a) Calculated electronic band gaps as a function of pressure for *K*_4_-CH, Hex-CH, *T*_8_-CH, and *R*_6_-CH. (b) and (c) Absorption spectra along the *c* axis and in the *ab* plane for *T*_8_-CH and *R*_6_-CH at 0, 30, and 50 GPa, respectively.

The electronic band structure calculations within the hybrid functional method^36^ have demonstrated the insulating nature of the two chiral saturated CH phases. The valence band maximums of *T*_8_-CH [Fig. 3(c)] and *R*_6_-CH [Fig. 3(d)] are at the Γ and A points in the Brillouin zone while the conduction band minimums are along the Z-X direction and at the M point, giving indirect band gaps of 6.67 and 5.88 eV at 0 GPa, respectively. Hence, both chiral phases are predicted to be optically transparent as previously proposed *K*_4_-CH (6.07 eV) and Hex-CH (6.54 eV). We have further explored the electronic properties at increasing pressures. The calculated band gaps as a function of pressure for *K*_4_-CH, Hex-CH, *T*_8_-CH, and *R*_6_-CH are shown in Fig. 4(a). According to the results, *T*_8_-CH remains dielectric at pressures up to at least 50 GPa. The band gap has only a weak dependence on pressure and decreases from 6.67 to 6.42 eV as pressure increases from 0 to 50 GPa. For *R*_6_-CH, the band gap decreases more rapidly with increasing pressure and reaches 88% of the original band gap at 50 GPa, indicating a stronger pressure dependence similar to those for *K*_4_-CH and Hex-CH.

We now move on to discuss the optical properties of the *T*_8_-CH and *R*_6_-CH phases in terms of the calculated frequency dependent imaginary part of the dielectric matrix^37^. By comparing the optical spectra at 0 GPa obtained with light polarized either along the *c* axis or in the *ab* plane [Fig. 4(b) and 4(c)], we find that the spectra differ in shape and intensity, suggesting anisotropic features for both chiral phases. The application of pressure on the two structures induces an opposite optical response. By increasing pressure the C-C bond length is shortened, the band gap decreases and the whole optical spectrum is almost rigidly shifted toward higher energy. The calculated static dielectric constants for the two chiral phases increase slowly with pressure, with the *ab*- (*c*-) components going from 4.045 (4.071) and 3.846 (3.797) at 0 GPa to 4.174 (4.227) and 4.082 (3.934) at 30 GPa for *T*_8_-CH and *R*_6_-CH, respectively.

**Figure 5:**
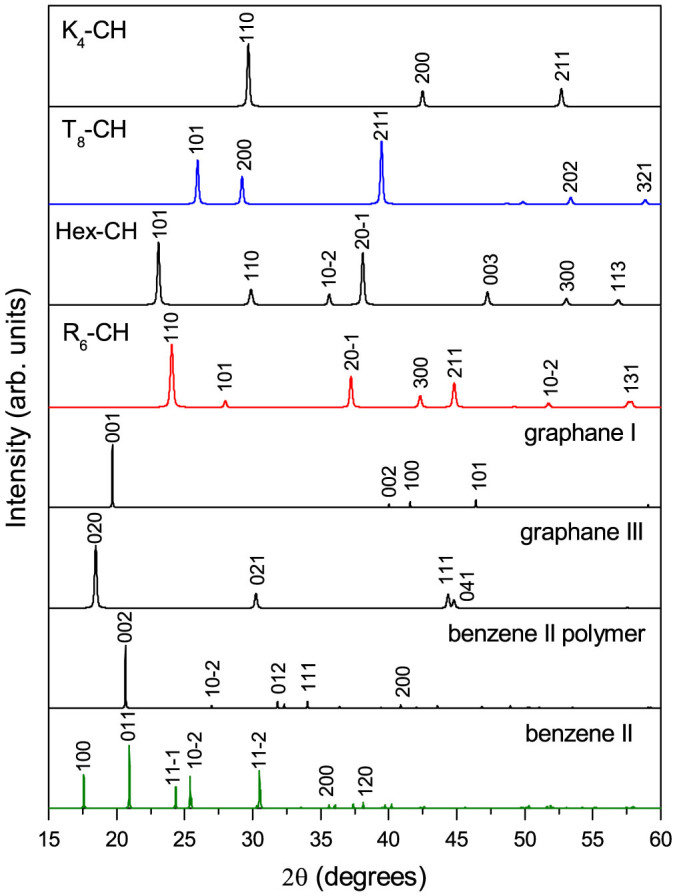
Simulated XRD patterns for *K*_4_-CH, Hex-CH, *T*_8_-CH, *R*_6_-CH, graphane I and III, benzene II polymer, and benzene II at 0 GPa. The x-ray wavelength is 1.540 56 Å.

To provide more information and characters for possible experimental observation, we have also simulated the x-ray diffraction (XRD) spectra of the various CH phases at 0 GPa. Monochromatic radiation with a wavelength 1.540 56 Å is used, and the results are shown in Fig. 5. Unlike *K*_4_-CH where the main peak (110) at 2*θ* = 29.69° is observed, three sharp XRD peaks of (101) at 25.95°, (200) at 29.23°, and (211) at 39.48° with strong intensities are seen for *T*_8_-CH. Furthermore, we also find significant difference between the two hexagonal phases, with the most prominent peak observed to be (101) at 23.08° for Hex-CH and (110) at 24.06° for *R*_6_-CH, although both phases share the same peak (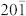) with close positions. Compared with these diamond-like CH phases, the layered ones have specific XRD peaks at relatively small 2*θ* values of 19.70° for graphane I, 18.46° for graphane III, and 20.65° for benzene II polymer, which originate from a large interlayer distance in layered crystal structure. We believe that the above comparison between the chiral hydrocarbons found here and other CH structures may be helpful for identifying them in experiments.

## Discussion

In summary, we have predicted by *ab initio* calculations two new 3D hydrocarbon framework structures composed of 3-fold and 4-fold helical CH chains in 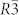 and *I*4_1_/*a* symmetry, respectively. These saturated crystalline phases are dynamically stable and have large bulk moduli of ~200 GPa. We confirm their increasing stabilization with pressure relative to the molecular solids such as acetylene, cubane, and benzene with 1:1 stoichiometry in the pressure range 0–50 GPa. Calculations on the electronic and optical properties show that both chiral hydrocarbons exhibit a wide-gap insulating behavior and anisotropic adsorption spectra. The simulated XRD patterns show the distinct structural feature with respect to the layered CH phases. The present results will stimulate future experiments on the high-pressure polymerization of molecular hydrocarbons to synthesize the amazing chiral CH phases, which may have wide applications in mechanical, optoelectronic, and biological materials.

## Methods

The calculations were performed using the density functional theory within the local density approximation (LDA)^29^,^30^ as implemented in the Vienna ab-initio simulation package (VASP)^31^. We adopted the projector augmented wave (PAW) method^32^ to describe the electron-ion interaction. The plane wave cutoff energy was set to 800 eV. Brillouin zone integration was carried out at Monkhorst-Pack^33^ k-point meshes with a grid spacing of 2*π* × 0.02 Å^−1^. The geometries were optimized by a conjugate gradient algorithm until the Hellmann-Feynman forces on the ions are less than 10^−3^ eV/Å. Phonon calculations were based on the supercell approach^34^ using the PHONOPY code^35^. The HSE06 hybrid functional method^36^ was employed to calculate the electronic and optical properties. The frequency dependent dielectric matrix was obtained by neglecting local field effects^37^.

## Author Contributions

C.S.L., H.D.L. and J.T.W. designed the study and wrote the paper; C.S.L. carried out *ab initio* simulations. All authors discussed the results and contributed to the manuscript.

## Figures and Tables

**Table 1:** Calculated equilibrium structural parameters (space group, lattice parameters *a*, *b*, and *c*, volume V_0_, bond lengths d*_C_*_–*C*_), total energy E*_tot_*, bulk modulus *B*_0_, and electronic band gap E*_g_* for various hydrocarbon phases at zero pressure. Energies are given relative to that of graphane I. For benzene II and benzene II polymer, *β* = 110.62 and 97.49°, respectively. The C-H bond lengths are around 1.10 Å for all phases

Structure	Space group	*a*, *b* (Å)	*c* (Å)	V_0_ (Å^3^/CH)	d*_C_*_–*C*_ (Å)	E*_tot_* (eV/CH)	B_0_ (GPa)	E*_g_* (eV)
*K*_4_-CH	*I*2_1_3	4.252		9.62	1.565	0.43	198.4	6.07
Hex-CH	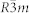	5.974	5.764	9.92	1.542, 1.575	0.27	175.2	6.54
*T*_8_-CH	*I*4_1_/*a*	6.106	4.146	9.67	1.559, 1.564	0.43	201.7	6.67
*R*_6_-CH	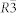	7.392	3.671	9.66	1.543, 1.561	0.30	185.2	5.88
Benzene II	*P*2_1_/*c*	5.382, 5.338	7.457	16.96	1.387	0.32	9.8	5.14
Solid cubane	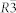	5.918	10.991	14.07	1.557	0.90	18.9	6.60
Solid acetylene	*Cmca*	4.978, 5.713	5.742	20.49	1.208	1.59	9.1	5.43
Benzene II polymer	*P*2_1_/*c*	4.449, 3.713	8.667	12.53	1.517–1.551	0.34	31.3	5.05
Graphane III	*Cmca*	2.515, 9.502	3.745	11.51	1.524, 1.522	0.04	30.6	4.76
Graphane I	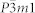	2.506	4.503	12.41	1.515	0.00	19.7	4.87
